# Two new species and three new records of *Diachea* (Physarales) from China based on morphological and molecular evidence

**DOI:** 10.3389/fmicb.2024.1458944

**Published:** 2024-11-06

**Authors:** Xuefei Li, Dan Dai, Yonglan Tuo, You Li, Jiajun Hu, Frederick Leo Sossah, Bo Zhang, Xiao Li, Yu Li

**Affiliations:** ^1^Engineering Research Center of Chinese Ministry of Education for Edible and Medicinal Fungi, Jilin Agricultural University, Changchun, China; ^2^College of Mycology, Jilin Agricultural University, Changchun, China; ^3^Institute of Agricultural Applied Microbiology, Jiangxi Academy of Agricultural Sciences, Nanchang, China; ^4^School of Life Science, Zhejiang Normal University, Jinhua, China; ^5^Council for Scientific and Industrial Research (CSIR), Oil Palm Research Institute, Coconut Research Programme, Sekondi, Ghana; ^6^Industrial Development Institute for Plants, Animals, and Fungi Integration of Biyang County, Zhumadian, China

**Keywords:** diversity, morphology, multigene phylogeny, new taxa, taxonomy

## Abstract

*Diachea* is an important genus of myxomycetes, recognized for its ecological role and wide distribution. This study aimed to expand knowledge of species diversity within this genus in China. We collected *Diachea* specimens from various locations in Shaanxi and Sichuan provinces and characterized them through morphological analysis and phylogenetic analysis using four genetic markers: small subunit ribosomal RNA (nSSU), translation elongation factor 1-alpha (*EF-1α*), mitochondrial small subunit (mtSSU), and alpha-tubulin gene (*α-Tub*). Based on these analyses, we describe two new species, namely, *Diachea plectophylla* and *D. sichuanensis*, discovered in the Shaanxi and Sichuan provinces, respectively. *Diachea plectophylla* is distinguished by its dense, rigid capillitium, spore warts, and distinct separation of capillitium ends from the peridium. *Diachea sichuanensis*, closely related to *D. leucopodia*, is identified by its blunt-headed columella, clustered spore warts, and robust stalks. In addition to these new species, we recorded five previously documented species, including *D*. *bulbillosa* in Gansu province, *D. leucopodia* in Yunnan and Sichuan provinces, and *D. subsessilis* in Sichuan province. Detailed descriptions, micrographs, taxonomic comparisons, and an identification key are provided to aid in accurate identification. The discovery of these new species not only enhances the known diversity of slime molds in the region but also provides valuable information for future studies on their geographical distribution and ecological relationships.

## 1 Introduction

Myxogastria, commonly known as true slime molds, are fungal-like eukaryotes classified within the kingdom Protozoa, forming a monophyletic group within the Amoebozoa (Adl et al., 2012; Kang et al., 2017). These organisms typically thrive in damp, cool environments, obtaining nutrients from decaying substrates such as rotting wood, dead leaves, branches, and moss. While myxomycetes can adapt to various vegetation types and environmental conditions, they show a preference for these specific habitats. According to Lado (2005/2024), over 1,100 species of myxomycetes are currently recognized.

The genus *Diachea* Fr., within the order Physarales, includes only 18 known species worldwide, seven of which have been reported in China. Species within this genus are primarily distinguished by the glittering gold, bronze, or bluish iridescence of their peridium. The capillitium, a network of threads inside the sporotheca, lacks limestone and is dark brown. Both the stalk and columella, a prominent structure at the base of the sporotheca or plasmodiocarp, contain granular or crystalline limestone (Martin and Alexopoulos, 1969; Keller et al., 2004; Kirk et al., 2008; Poulain et al., 2011).

*Diachea* was established by Fries in 1825 based on *D. leucopodia* (Bull.) Rostaf. (Martin and Alexopoulos, 1969). Historically, researchers have focused on species within *Diachea* that featured a halo-peridium, capillitium without limestone, and a well-developed columella—traits resembling those of the Stemonitidales. As a result, *Diachea* was initially classified in the family *Stemonitaceae*, suggesting a close relationship with *Lamproderma* (Fries, 1829; Martin and Alexopoulos, 1969). However, resemblances to species in the Physarales prompted Rostafiński (1874) and Alexopoulos and Saenz (1975) to reclassify it into the family *Didymiaceae* within the order Physarales (Alexopoulos and Saenz, 1975). Subsequent research by Cavalcanti et al. (2006) supported the placement of *D. leucopodia* within *Didymiaceae* and Physarales.

The classification of *Diachea* has fluctuated between the Physarales and Stemonitidales (Alexopoulos, 1982; Kalyanasundaram and Mubarak, 1989; Lado, 2005/2024). Recent studies by García-García-Martín et al. (2023) and Prikhodko et al. (2023), using multigene phylogenetic analyses, confirmed that *Diachea* belongs to *Didymiaceae* within Physarales. This taxonomic uncertainty may stem from variations in capillitium features during different developmental stages. A comprehensive phylogenetic analysis of *Diachea* remains incomplete due to limited sequence data, leaving its classification unresolved.

China’s diverse natural environment contributes to a rich myxomycetes species diversity. However, research on this group in China remains limited. In this study, we describe two new *Diachea* species and confirm the presence of five previously recorded species. We employed four genetic markers (nSSU, *EF-1α*, mtSSU, and *α-Tub*) for our molecular analyses. The discovery of these new and newly recorded species enhances our understanding of myxomycetes diversity and provides essential data for future systematic studies of the genus *Diachea*.

## 2 Materials and methods

### 2.1 Morphological studies

Specimens were collected from eight locations across China over a 12-year period (2010–2021). Samples, including dead bark, leaves, branches, and litter, were gathered in the field, air-dried in a ventilated area, and stored in collection boxes. For species description, photographs of mature sporocarps were taken using a Zeiss dissecting microscope (Axio Zoom V16, Carl Zeiss Microscopy GmbH, Göttingen, Germany) and a Leica stereoscopic dissector (Leica M165, Wetzlar, Germany). Light micrographs were captured with a Lab A1 microscope (Carl Zeiss AG, Jena, Germany) and processed using ZEN 2.3 software (Carl Zeiss AG). Dried specimens were mounted in 3% KOH for spore examination under oil immersion, and the diameters of 20 spores were measured. Spore and capillitium dimensions are reported as (minimum–) 25th percentile – 75th percentile (−maximum) (Leontyev et al., 2023). Specimens were also examined using a scanning electron microscope (SEM), and photomicrographs were taken using a Hitachi S-4800 SEM (Japan) at 10–15 kV. All analyzed specimens were deposited in the myxomycete collection of the Herbarium of Mycology at Jilin Agriculture University (HMJAU), China.

### 2.2 DNA extraction, amplification, and sequencing

Genomic DNA from the putative new species was extracted from dried materials using the TIANamp Micro DNA Kit (TianGen Biotech Co., Ltd., Beijing, China). Four genetic markers were used: nSSU (S2F/SR4DarkR), *EF-1α* (PB1F/PB1R), mtSSU (Kmit-F/Kmit-R), and *α-Tub* (kTub-F2/KTub-R1) (García-Martín et al., 2023; Novozhilov et al., 2013). These markers are commonly used for barcoding in protists (Adl et al., 2014) and myxomycetes (García-Cunchillos et al., 2022; Leontyev and Schnittler, 2022; García-Martín et al., 2023). The PCR mixture composition, sample preparation, and sequencing protocols followed Prikhodko et al. (2023) and García-Martín et al. (2023). PCR products were analyzed via gel electrophoresis and purified using the Universal DNA Purification Kit (TianGen Biotech Co., Ltd., Beijing, China). Purified PCR products were sequenced by Kumei Biotechnology Co., Ltd. (Changchun City, China) using the Sanger method. The resulting sequences were deposited in the National Center for Biotechnology Information (NCBI) GenBank database (Table 1).^1^

**Table 1:** Information for the sequences used in this study.

Scientific name	Voucher/specimen numbers	GenBank accession numbers				Reference
		nSSU	*EF-1α*	mtSSU	*α-Tub*	
*Diachea bulbillosa*	MM32108	–	–	ON059548	–	Lado et al. (2022)
***D. bulbillosa***	**HMJAU M10037**	**PP795178**	**PP796508**	**PP833125**	–	**This study**
***D. bulbillosa***	**HMJAU M10002**	**PP795179**	**PP796509**	**PP833126**	–	**This study**
*D. cylindrica*	MM46123	ON059420	–	ON059549	–	Lado et al. (2022)
*D. cylindrica*	TVDH548	–	ON081604	ON059550	OP630984	Lado et al. (2022); García-Martín et al. (2023)
*D. dictyospora*	MM39407	OP646293	OP631018	OP646253	OP630979	García-Martín et al. (2023)
***D. dictyospora***	**HMJAU M20124-2**	**PP795180**	**PP796510**	**PP833127**	–	**This study**
***D. dictyospora***	**HMJAU M20124-1**	**PP795181**	**PP796511**	**PP833128**	–	**This study**
*D. leucopodia* s. lat.	MCCNNU00043	KF743861	KF743855	–	–	Unpublished
*D. leucopodia* s. lat.	MCCNNU00134	KF743862	KF743856	–	–	Unpublished
*D. leucopodia* s. lat.	MA-Fungi 90,990	MG963646	MG963478	MG963551	MG963709	García-Martín et al. (2023)
*D. leucopodia* s. lat.	MA-Fungi 68,824	–	ON081605	ON059551	OP630985	Lado et al. (2022)
*D. leucopodia* s. lat.	MA-Fungi 86,466	MG963645	MG963477	MG963550	MG963708	García-Martín et al. (2023)
*D. leucopodia* s. lat.	MA-Fungi 86,465	MG963644	MG963476	MG963549	MG963707	García-Martín et al. (2023)
***D. leucopodia s. lat.***	**HMJAU M10086**	**PP795182**	**PP796512**	**PP833129**	**PP796523**	**This study**
***D. leucopodia s. lat.***	**HMJAU M10005**	**PP795183**	**PP796513**	**PP833130**	**PP796524**	**This study**
***D. macroverrucosa***	**HMJAU M10039**	**PP795184**	**PP796514**	**PP833131**	**–**	**This study**
***D. macroverrucosa***	**HMJAU M10067**	**PP795185**	**PP796515**	**PP833132**	**–**	**This study**
***D. macroverrucosa***	**HMJAU M10064**	**PP795186**	**PP796516**	**PP833133**	**–**	**This study**
*D. mitchellii*	MA-Fungi 96,696	ON059424	ON081610	ON059556	–	Lado et al. (2022)
*D. mitchellii*	MA-Fungi 91,212	ON059421	ON081607	ON059553	OP630986	Lado et al. (2022)
*D. mitchellii*	MA-Fungi 96,407	ON059423	ON081609	ON059555	OP630987	Lado et al. (2022)
*D. obovata*	MM47920	OP646297	OP631019	OP646257	OP630983	García-Martín et al. (2023)
*D. obovata*	MM39542	OP646296	–	OP646256	OP630982	García-Martín et al. (2023)
*D. obovata*	MM37196	OP646295	–	OP646255	OP630981	García-Martín et al. (2023)
***D. plectophylla***	**HMJAU M10006**	**PP795187**	**PP796517**	**PP833134**	**–**	**This study**
***D. plectophylla***	**HMJAU M10004**	**PP795188**	**PP796518**	**PP833135**	**–**	**This study**
***D. sichuanensis***	**HMJAU M10022**	**PP795189**	**PP796519**	**PP833136**	**–**	**This study**
***D. sichuanensis***	**HMJAU M10044**	**PP795190**	**PP796520**	**PP833137**	**–**	**This study**
*D. silvaepluvialis*	MA_Fungi_51360	ON059425	–	ON059558	–	Lado et al. (2022)
*D. silvaepluvialis*	MA_Fungi_51896	–	MW240029	MW240161	–	García-Martín et al. (2023)
*D. subsessilis*	MA_Fungi_60512	MW240302	MW240030	OP646258	OP630988	García-Martín et al. (2023)
*D. subsessilis*	MA_Fungi_82029	ON059427	–	ON059561	–	Lado et al. (2022)
*D. subsessilis*	MA_Fungi_68835	ON059426	–	ON059560	–	Lado et al. (2022)
***D. subsessilis***	**HMJAU M10075**	**PP795191**	**PP796521**	**PP833138**	**–**	**This study**
***D. subsessilis***	**HMJAU M10032**	**PP795192**	**PP796522**	**PP833139**	**–**	**This study**
*Didymium dubium*	MA-Fungi 63,904	MW240326	MW240058	MW240188	MW239915	García-Martín et al. (2023)
*D. dubium*	MA-Fungi 80,036	MW240327	MW240059	MW240189	MW239916	García-Martín et al. (2023)
*D. dubium*	MA-Fungi 80,492	MG662512	MW240060	MW240190	MW239917	García-Martín et al. (2023)
*D. dubium*	K7	AM231294	–	–		Wikmark et al. (2007)
*D. melanospermum*	MA-Fungi 91,238	MG963668	MG963497	MG963577	MG963736	García-Martín et al. (2023)
*D. melanospermum*	MA-Fungi 62,790	MG963667	MW240068	MG963576	MG963735	García-Martín et al. (2023)
*D. nivicola*	MA-Fungi 90,573	MT227090	MT230925	–	–	Janik et al. (2020)
*D. nivicola*	AH19667	MT227019	MT230908	–	–	Janik et al. (2020)
*D. yulii*	HMJAU M3002	MF149871	MK905755	–	–	Zhao et al. (2021)
*D. yulii*	HMJAU M3001	MF149870	MK905754	–	–	Zhao et al. (2021)
*Echinostelium coelocephalum*	ATCC MYA 2964	AY842033	AY643813	–	–	Fiore-Donno et al. (2005)
*E. minutum*	ATCC 22345	AY842034	AY643814	–	–	Fiore-Donno et al. (2005)
*Enerthenema intermedium*	MM 21635	DQ903688	–	–		Fiore-Donno et al. (2008)
*E. melanospermum*	MM 28388	DQ903689	–	–		Fiore-Donno et al. (2008)
*E. papillatum*	AMFD141	AY643823	–	–		Fiore-Donno et al. (2005)
*Lamproderma aeneum*	MA-Fungi 81,947	MW240352	MW240092	MW240217	MW239949	García-Martín et al. (2023)
*L. aeneum*	MA-Fungi 86,925	MW240353	MW240093	MW240218	–	García-Martín et al. (2023)
*L. aeneum*	MA-Fungi 90,422	MW240354	MW240094	MW240219	–	García-Martín et al. (2023)
*L. ovoideum*	Sc30802	MN595543	MN596918	–	–	Shchepin et al. (2024)
*Macbrideola oblonga*	M. Schnittler	DQ903682	–	–	–	Fiore-Donno et al. (2008)
*Meriderma aggregatum*	AMFD135	DQ903669	–	–	–	Fiore-Donno et al. (2008)
*M. carestiae* var. *retisporum*	AMFD173	DQ903671	–	–	–	Fiore-Donno et al. (2008)
*M. fuscatum*	MM 24907	DQ903668	–	–	–	Fiore-Donno et al. (2008)
*Nannengaella globulifera*	MA-Fungi 51,647	MF352479	MF352528	MW240269	MW239991	García-Martín et al. (2018)
*N. globulifera*	MA-Fungi 46,711	MW240379	–	MW240268	MW239990	García-Martín et al. (2023)
*N. globulifera*	MYX8635	MW693010	MW701657	–	–	Shchepin et al. (2022)
*N. globulifera*	MYX8695	MW693014	MW701661	–	–	Shchepin et al. (2022)
*N. mellea*	MA-Fungi 69,850	MF352484	MF352534	MG963629	MG963776	García-Martín et al. (2018)
*N. mellea*	MA-Fungi 87,986	MF352485	MF352535	MG963630	MG963777	García-Martín et al. (2018)
*N. mellea*	MA-Fungi 60,322	MW240383	MG963528	MG963628	MG963775	García-Martín et al. (2023)
*Physarum didermoides*	MA-Fungi 71,195	MW240378	OP650825	MW240267	–	García-Martín et al. (2023)
*P. didermoides*	MA-Fungi 57,262	MF352488	MF352542	MW240266	MW239988	García-Martín et al. (2018)
*P. leucophaeum*	MA-Fungi 49,730	MG963686	MG963521	MG963614	MW239996	García-Martín et al. (2023)
*P. leucophaeum*	MA-Fungi 78,861	MG963688	–	MG963618	MG963767	García-Martín et al. (2023)
*P. leucophaeum*	MA-Fungi 59,323	MF352477	MF352526	MG963615	MG963765	García-Martín et al. (2018)
*P. straminipes*	MA-Fungi 70,363	MF352489	MF352543	MW240291	MW240015	García-Martín et al. (2018)
*P. straminipes*	MA-Fungi 87,865	MW240394	–	MW240292	MW240016	García-Martín et al. (2023)
*P. viride*	LE302489	MW693022	MW701670	–	–	Shchepin et al. (2022)
*P. viride*	LE317322	MW693024	MW701672	OP616654	–	Shchepin et al. (2022); Prikhodko et al. (2023)
*Symphytocarpus impexus*	–	AY230188	–	–		Walkera et al. (2003)

Sequences produced in this study are in bold.

### 2.3 Phylogenetic analysis

For phylogenetic analysis, we used 214 sequences derived from 33 species to construct a concatenated dataset. Raw sequence editing and assembly were performed using BioEdit 7.1.3 (Hall, 1999). Sequences for nSSU, *EF-1α*, mtSSU, and *α-Tub* from GenBank were retrieved for other *Diachea* species, as well as representatives from the families *Didymiaceae* and *Physaraceae*. Single-gene alignments were generated using MAFFT V7.490 (Katoh and Standley, 2013), and manual adjustments were made in BioEdit (Hall, 1999). Phylogenetic analyses were performed separately for each gene and for the concatenated dataset using PhyloSuite v1.2.2 (Zhang et al., 2020; Xiang et al., 2023).

Maximum likelihood (ML) and Bayesian inference (BI) methods were employed for phylogenetic tree construction. ML analyses were conducted using IQ-TREE 1.6.12 with 1,000 non-parametric replicates (Nguyen et al., 2015). BI analyses were performed with MrBayes 3.2.6 (Ronquist and Huelsenbeck, 2003), running two simultaneous chains for 10 million generations; trees were sampled every 1,000 generations, and the first 25% were discarded as burn-in. Convergence of the Markov chain Monte Carlo (MCMC) was assessed with TRACER 1.7.2 (Rambaut et al., 2018). Phylogenetic trees were visualized using ITOL version 6.9 (Bork, 2007) and further edited using Adobe Illustrator (San Jose, CA, United States).

## 3 Results

### 3.1 Phylogenetic analyses

We analyzed 167 sequences representing 33 species in this study, including 47 newly acquired sequences from the specimens examined: 15 sequences each for nSSU, *EF-1α*, and mtSSU and 2 sequences for *α-Tub*. The dataset consisted of 72 sequences with 7,606 characters for nSSU, 57 sequences with 2,505 characters for *EF-1α*, 55 sequences with 477 characters for mtSSU, and 29 sequences with 1,007 characters for *α-Tub*, including gaps.

**Figure 1:**
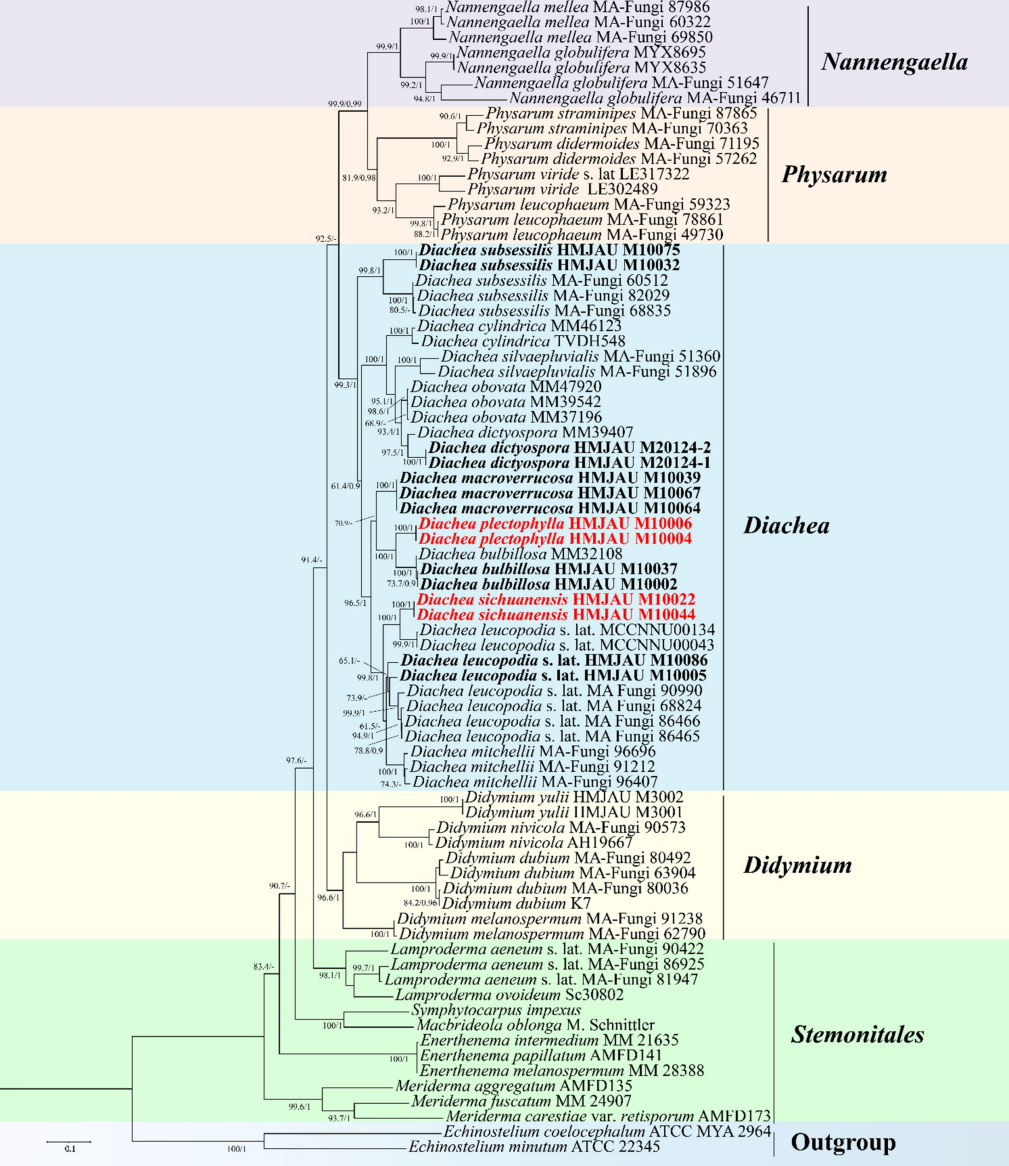
Maximum likelihood phylogenetic tree generated from the nSSU, *EF-1α*, mt SSU, and *α-Tub* datasets of the target clade containing the new species from *Diachea*. The two values of internal nodes, respectively, represent the maximum likelihood bootstrap (BS)/Bayesian posterior probability (PP). This study species is in bold and red font.

The phylogenetic trees generated using both maximum likelihood (ML) and Bayesian inference (BI) analyses showed consistent topologies across the single-gene datasets. For clarity, only the ML tree is presented here. In the multigene phylogenetic tree, which combines the alignments of nSSU, *EF-1α*, mtSSU, and *α-Tub* sequences, all 11 specimens of the genus *Diachea* formed a cohesive and well-supported clade (PP = 1, BS = 99.3%). Within this group, *Diachea plectophylla* and *D. sichuanensis* emerged as distinct, strongly supported clades (PP = 1, BS = 100%). These results, along with unique morphological traits, confirm the discovery of these two previously undocumented species, identifying *D*. *plectophylla* and *D. sichuanensis* as distinct lineages (Figure 1).

**Taxonomy**

***Diachea plectophylla* X.F. Li, D. Dai, B. Zhang & Y. Li, sp. nov.**

MycoBank: MB854209.

(Figure 2)

**Figure 2:**
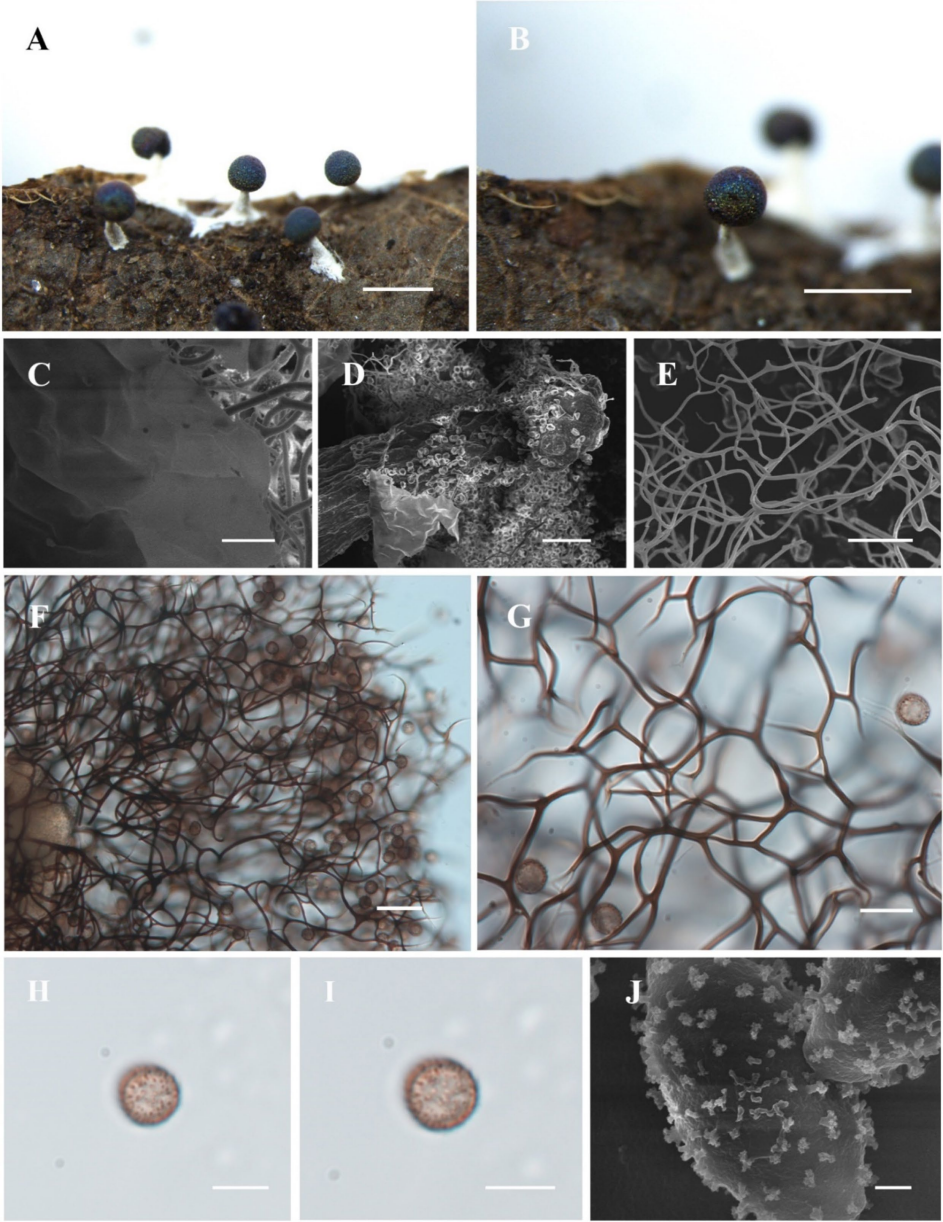
Habitat and microstructure of *Diachea plectophylla* (HMJAU M10004). (A,B) Sporocarps; (C) peridium by SEM; (D) columella by SEM; (E–G) capillitium by SEM and TL; (H–J) spores with cluster warts by TL and SEM. Scale bars: (A), B = 1 mm, C = 10 μm, (D), *F* = 40 μm, (E), G = 20 μm, H, I = 10 μm, J = 1 μm.

**Diagnosis.** Capillitium dense, hard, and straight; ends not connected to the peridium.

**Holotype.** China, Shaanxi province, Niubeiliang National Nature Reserve, on the rotten leaves, 23 July 2014, B. Zhang, HMJAU M10004.

**Etymology.** The epithet “*plectophylla*” refers to crude the dense capillitium.

**Description.** Sporocarp, gregarious, stipitate, spherical, with blue-purple halo, (0.3–) 0.35–0.5 (−0.55) mm in diameter, (0.5–) 0.6–1.2 (−1.3) mm in tall. Stalk calcareous, white, with enlarged at the base, tapering upward, with numerous calcareous particles. Peridium single-layered, thin, halo, persistent, irregular dehiscence. Hypothallus inconspicuous. Columella white, conical, blunt-headed, tapering upward, reaching approximately half of the sporotheca, an extension of the stalk. Capillitium dense, thick, hard, black brown, branched, and connected, radiating from columella, not connected to the peridium. Spores dark in mass, dark brown by transmitted light, with sparse warts, sometimes warts connect to form ridges, 8–9 (−9.5) μm in diameter.

**Habitat.** On the rotten leaves and branches.

**Distribution in China.** Shaanxi province.

**Distribution in the World.** China.

**Additional specimens examined.** China, Shaanxi province, Niubeiliang National Nature Reserve, on the rotten leaves, 23 July 2014, B. Zhang, HMJAU M10006.

**Notes.** *Diachea plectophylla* is morphologically similar to *D. bulbillosa*, *D. macroverrucosa*, and *D. splendens* due to its blue halo, subglobose sporocarp, white stalk, and obvious columella (Table 2). However, *D. plectophylla* differs from *D. macroverrucosa* in having relatively large columella and small spores. *Diachea plectophylla* differs from *D. bulbillosa* in its dense capillitium and small spores (Table 2). *Diachea plectophylla* can be distinguished from *D. splendens* by its dense capillitium, where warts connect to form ridges, whereas *D. splendens* has sparse reticular capillitium, and spores have rough nodules and ridges, arranged in a network pattern. From the phylogenetic tree, *D. plectophylla* is similar to *D. bulbillosa* but has a separate branch.

**Table 2:** Comparison of morphological characteristics of *Diachea* species in this study.

	Sporocarps	Stalk	Peridium	Hypothallus	Columella	Capillitium	Spores
*D. plectophylla*	Spherical, with blue-purple halo, (0.3–) 0.35–0.5 (−0.55) mm in diameter, (0.5–) 0.6–1.2 (−1.3) mm in tall.	Stalk calcareous, white	Single-layered, thin, halo, persistent, dehiscence irregular.	Inconspicuous	White, conical, blunt-headed, reaching approximately half of the sporotheca.	Black brown, radiating from various parts of the columella, not connected to the peridium.	With sparse warts, 8–9 (−9.5) μm in diameter.
*D. sichuanensis*	Cylindrical, metal blue, sometimes purple or bronze halo, blunt-headed, (0.35–) 0.4–0.6 (−0.65) mm in diameter, (0.9–) 1–2 (−2.2) mm in tall.	Stalk calcareous, brittle, snow-white, 1/4–1/2 of full height.	Single-layered, thin, halo, persistent, dehiscence irregular.	Conspicuous, calcareous, white.	White, thick, blunt-headed, calcareous, often close to the top of the sporotheca.	Brown, light-colored at the end, extending from various parts of the columella.	With clustered warts, (7–) 8–11 μm in diameter.
*D. dictyospora*	Spherical, upper white, lower brown, (0.3–) 0.35–0.4 (−0.45) mm in diameter, (1–) 1.2–1.4 (−1.5) mm in tall.	Stalk calcareous, yellow brown, dark brown at the top.	Single-layered, white or gray, slightly halo, the lower part persistent in a reddish-brown	Membranous, transparent, light brown.	Light brown, rod-shaped, and grows to the center of the sporotheca.	Sparse, lime nodes large, white, presenting a network within the sporotheca.	With obvious coarse warts, 12–13.5 (−16) μm in diameter.
*D. bulbillosa*	Spherical or subglobose, with blue or purple halo, (0.3–) 0.35–0.5 (−0.55) mm in diameter, (0.8–) 0.9–1.3 (−1.4) mm in tall.	Stalk cylindrical, calcareous, white.	Single-layered, with halo, irregularly cracked.	Inconspicuous, calcareous, white.	White, with an enlarged tip, club-shaped, generally reaching half or more of the sporotheca.	Purple-brown, curved, reticulate.	With sparsely and irregularly warts, (7–) 8–11 μm in diameter.
*D. leucopodia*	Cylindrical or elliptical, metal blue, or purple or bronze halo, (0.35–) 0.4–0.6 (−0.65) mm in diameter, (0.8–) 1–2 (−2.2) mm in tall.	Stalk slender, calcareous, snow-white, 1/2 of full height.	Single-layered, often falls off and dehiscence irregularly.	White, calcareous, with veins and often forming a network.	White, coniform, calcareous, higher than half of the sporotheca.	Dark brown, colorless at the tips, radiate from various parts of the columella.	With minutely warted, 8–10 (−11) μm in diameter.
*D. macroverrucosa*	Spherical, haze and blue halo, (0.3–) 0.35–0.4 (−0.45) mm in diameter, (0.7–) 0.75–0.9 (−1) mm in tall.	Stalk coarse and short, (0.35–) 0.4–0.5 (−0.55) mm in length, white, calcareous	Single-layered, with halo, dehiscence irregular.	Membranous, colorless and transparent, sometimes connected into sheets.	White, conical, blunt-headed, approximately 1/4 of the sporotheca.	Black brown, radiating from various parts of the columella, light-colored at the end, and not connected to the peridium.	With sparse dark warts, (8–) 9–13 μm in diameter.
*D. subsessilis*	Spherical, short-stalked or sessile, gray-green or dark blue halo, (0.35–) 0.4–0.8 (−0.85) mm in diameter, (0.5–) 0.6–1 (−1.2) mm in tall.	Stout, cylindrical, white, calcareous, not exceeding the height of the sporocarp.	Single-layered, with halo.	Network, small, slightly calcium or absent.	Short, calcareous, conical, sometimes absent.	Light brown, radiating from the columella, with light-colored at the end.	With clustered warts, (7–) 8–12 μm in diameter.

***Diachea sichuanensis* X.F. Li, D. Dai, B. Zhang & Y. Li, sp. nov.**

MycoBank: 854210.

Figure 3

**Figure 3:**
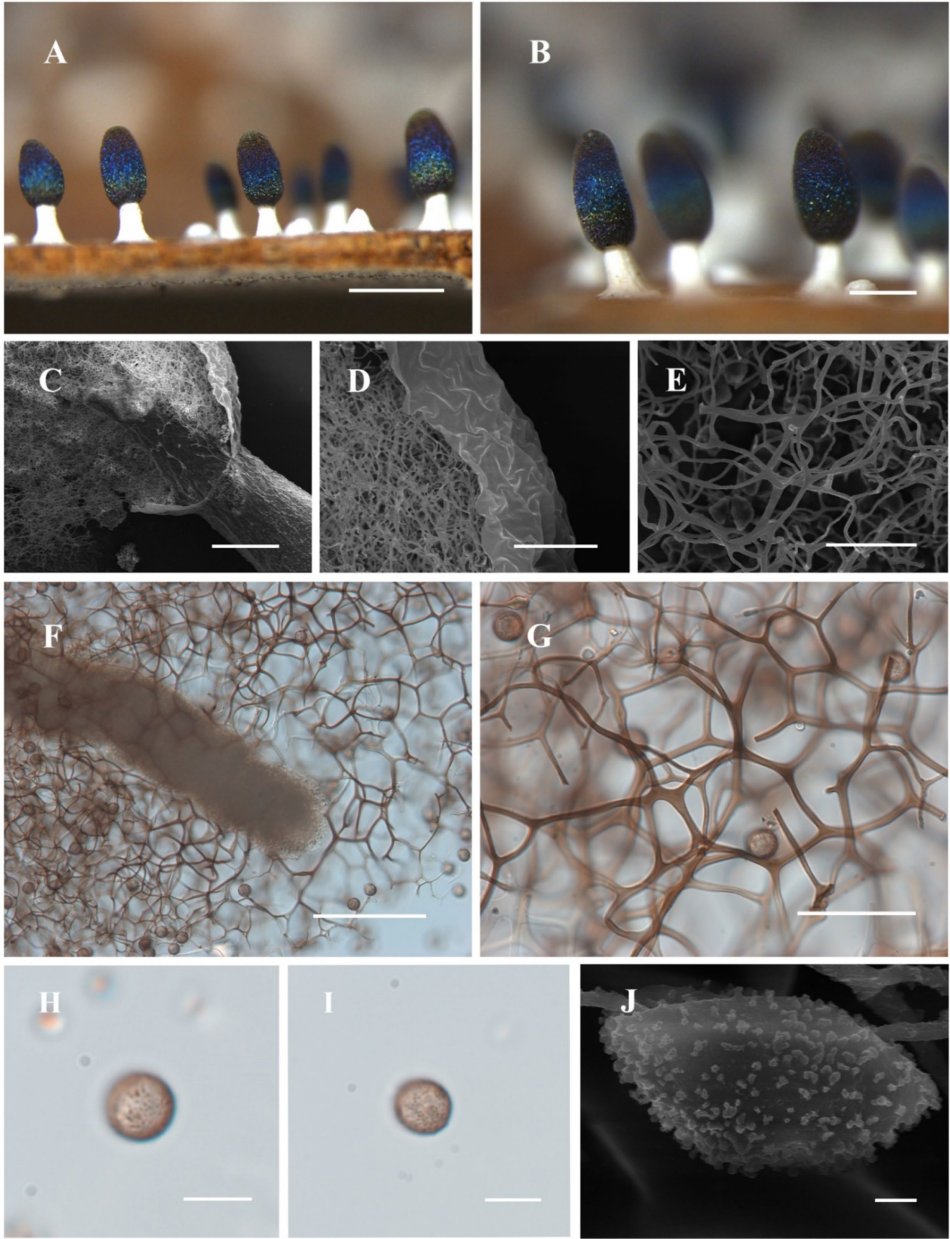
Habitat and microstructure of *Diachea sichuanensis* (HMJAU M10022). (A,B) Sporocarps; *C. columella* by SEM; (D,E) peridium and capillitium by SEM; (F) columella and capillitium by TL; (G) capillitium and spores by TL; (H–J) Spores with clustered warts by TL and SEM. Scale bars: A = 1 mm, B = 500 μm, C, *F* = 100 μm, D, G = 40 μm, E = 20 μm, H, I = 10 μm, J = 1 μm.

**Diagnosis**. Clustered warts are present on the surface of spores, and the capillitium is connected to the peridium.

**Holotype**. China, Sichuan province, Liangshan Yi Autonomous Prefecture, Mianning County, Lingshan Temple, on the branches, rotten leaves, and living plants, 12 July 2013, B. Zhang, HMJAU M10022.

**Etymology**. The epithet “*sichuanensis*” refers to Sichuan, the location of the holotype.

**Description**. Sporocarp dense, gregarious, stipitate, metal blue, sometimes with a purple or bronze halo, cylindrical or rarely subspherical, blunt-headed, (0.35–) 0.4–0.6 (−0.65) mm in diameter, (0.9–) 1–2 (−2.2) mm in tall. Stalk robust, calcareous, brittle, snow-white, 1/4–1/2 of full height, tapering upward. Peridium single-layered, thin, halo, persistent, with irregular dehiscence. Hypothallus conspicuous, white, calcareous, with a network of veins. Columella thick, gradually tapering upward, blunt-headed, white, calcareous, higher than half of the sporotheca, often close to the top of the sporotheca. Capillitium curved, branched, and connected, brown, light-colored at the end, radiating from various parts of the columella. Spore dark in mass, light purple under transmitted light, (7–) 8–11 μm in diameter, rough, with clustered warts.

**Habitat**. On the branches, rotten leaves, and living plants.

**Distribution in China**. Sichuan province.

**Distribution in the World**. China.

**Additional specimens examined**. China, Sichuan province, Liangshan Yi Autonomous Prefecture, Mianning County, Lingshan Temple, on the branches, rotten leaves, and living plants, 12 July 2013, B. Zhang, HMJAU M10044.

**Notes**. The primary characteristic of this species is the clustered warts on the surface of spores, with capillitium connected to the peridium. *Diachea sichuanensis* shares similarities with *D. leucopodia* in terms of sporocarp size and stalk color. However, *D. sichuanensis* can be distinguished from *D. leucopodia* by its blunt-headed columella, with spores of cluster warts and robust stalks. *Diachea sichuanensis* can be distinguished from *D. bulbillosa* by its spores with clustered warts, while the latter has sparse and large warts (Table 2). From the phylogenetic tree, *D. sichuanensis* is similar to *D. leucopodia*, but the genetic distance between the two is relatively far.

***Diachea dictyospora*** (Rostaf.) J.M. García-Martín, J.C. Zamora & Lado, *Persoonia* 51:106 (2023).

Figure 4

**Figure 4:**
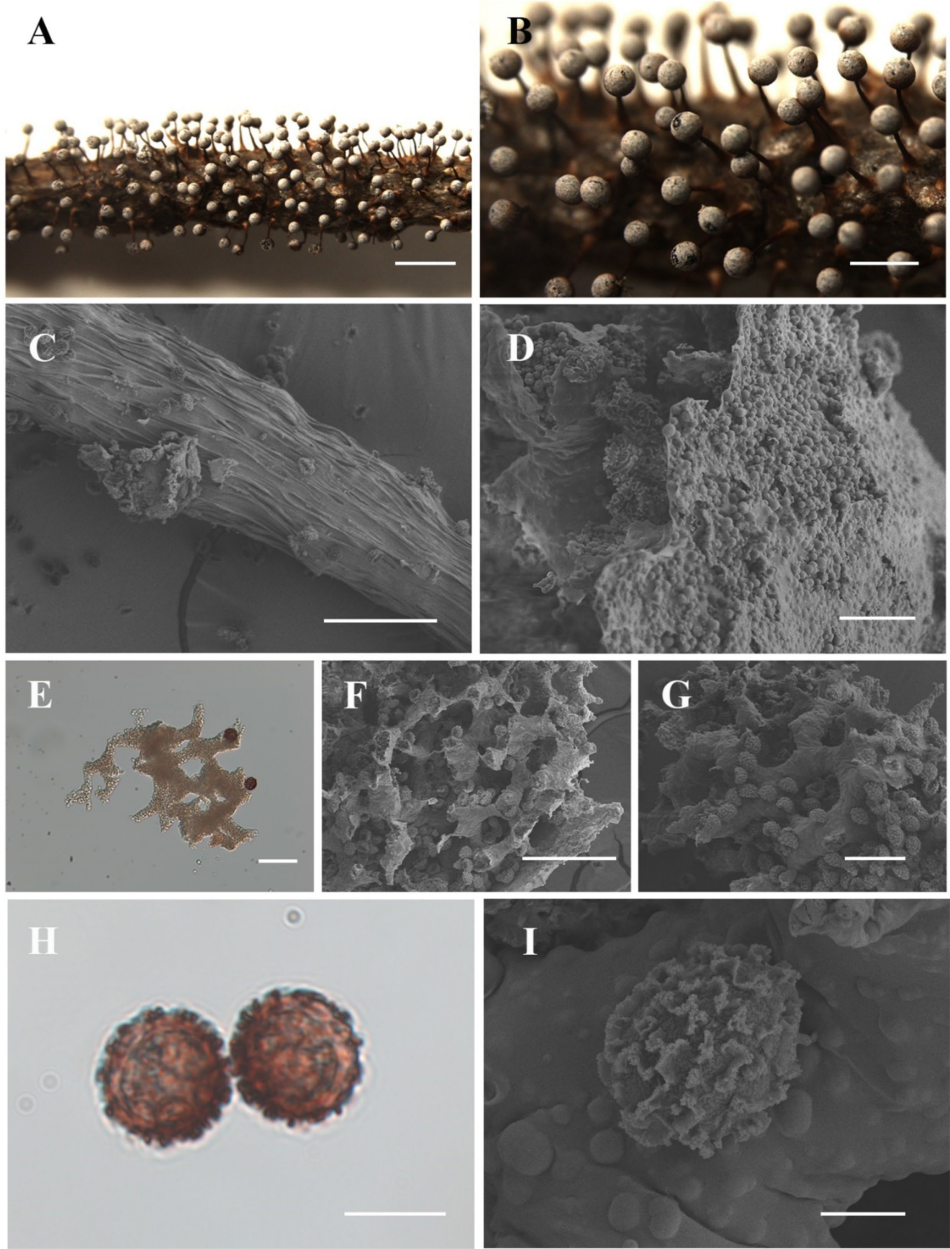
Habitat and microstructure of *Diachea dictyospora* (HMJAU M20124). (A,B). Sporocarps; (C) stalk by SEM; (D) peridium by SEM; (E) lime nodes by TL; (E–G) capillitium, lime nodes, and spores by SEM; (H–I) spores with sparse and large ridges by TL and SEM. Scale bars: A = 2 mm, B = 1 mm, C, F = 100 μm, D = 20 μm, E, G = 40 μm, H = 10 μm, I = 5 μm.

**Description.** Sporocarp, gregarious or densely crowded, erect, stipitate, spherical, upper white, lower brown, (0.3–) 0.35–0.4 (−0.45) mm in diameter, (1–) 1.2–1.4 (−1.5) mm in tall. Stalk slender, gradually tapering upward, calcareous, yellow brown, dark brown at the top, with longitudinal grooves, lighter, (0.7–) 0.8–1 (−1.1) mm in length. Peridium thin, membranous, irregularly cracked, covered with a layer of small calcareous particles on the surface, white or gray, slightly halo, with the upper part gray, the lower part persistent in a reddish-brown cup-shaped shape. Hypothallus membranous, transparent, light brown. Columella rod-shaped, light brown, and reaching the center of the sporotheca. Capillitium sparse, with large lime nodes, white, forming a network within the sporotheca. Spores dark in mass, red-brown by transmitted light, with coarse warts, sometimes forming an incomplete net of warts fused into shout ridge, 12–13.5 (−16) μm in diameter.

**Habitat.** On the dead branches.

**Distribution in China.** Jilin province and Heilongjiang province.

**Distribution in the World.** China, United States, Spain, France, Portugal, Germany, Italy, Japan.

**Specimens examined.** China, Jilin province, Baishan City, Fusong County, Lushuihe Town National Forest Park Hunting Ground, on the dead branches, 23 July 2018, B. Zhang, HMJAU M20124, HMJAU M20125, HMJAU M20126; China, Jilin province, Jian City, Wunvfeng National Forest Park, on the dead branches, 19 July 2018, B. Zhang, HMJAU M20127, HMJAU M20128.

**Notes.** The main characteristics of this species are red-brown or gray-brown sporocarp, large lime nodes, and dark, coarsely warted spores. *Diachea dictyospora* is similar to *D. muscorum* in terms of its sporocarp shape and spore color and size. However, some morphological features of *D. dictyospora* differ from *D. muscorum*, such as a short stalk and spores that have loosely reticulate ridges approximately 1 μm high. In contrast, *D. dictyospora* has a long stalk, with spores marked by an incomplete net of broad fused into short ridges. These two species previously belonged to the genus *Craterium* Trentep.; Lado et al. redefined the genus *Diachea* in 2023. *Diachea dictyospora*, *D. muscorum*, and *D. obovata* were transferred to this genus and classified based on their morphological characteristics (García-Martín et al., 2023).

***Diachea bulbillosa*** (Berk. & Broome) Lister, in Penzig, *Myxomyc. Fl. Buitenzorg* 47 (1898).

Figure 5

**Figure 5:**
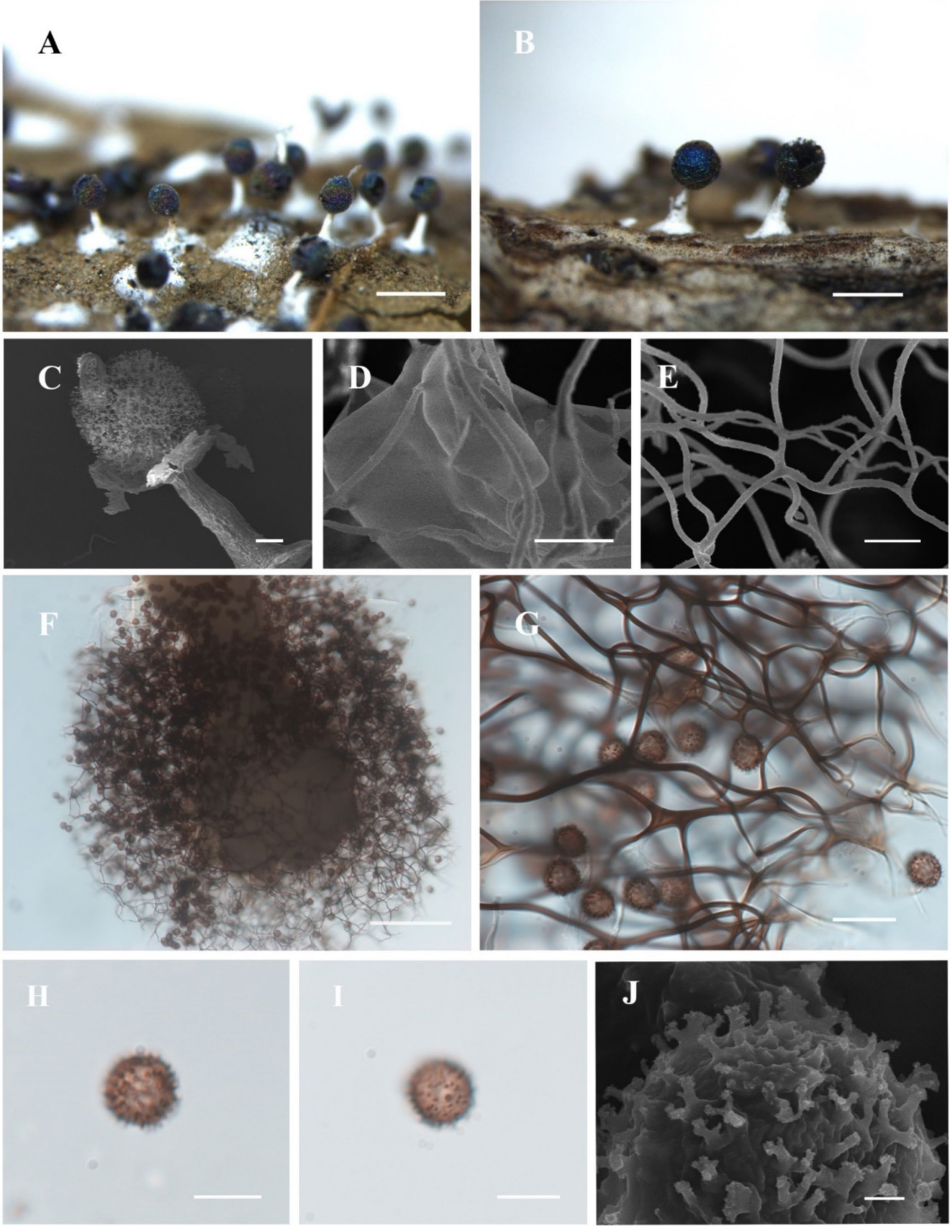
Habitat and microstructure of *Diachea bulbillosa* (HMJAU M10002). (A,B) Sporocarps; (C) sporocarp by SEM; (D) peridium and capillitium connected by SEM; (E) capillitium by SEM; (F) columella and capillitium by TL; (G) capillitium and spores by TL; H-J. Spores with sparse and large warts by TL. Scale bars: (A), B = 1 mm, (C), F = 100 μm, D, E = 5 μm, G = 20 μm, (H), I = 10 μm, J = 1 μm.

**Description.** Sporocarp, gregarious, stipitate, spherical or subglobose, with blue or purple halo, darkening with age, (0.3–) 0.35–0.5 (−0.55) mm in diameter, (0.8–) 0.9–1.3 (−1.4) mm in tall. Stalk cylindrical, gradually tapering upward, calcareous, white with an enlarged base. Peridium thin, membranous, with halo, irregularly cracked. Hypothallus inconspicuous, white. Columella obvious, white, calcareous, with an enlarged tip, club-shaped, reaching half or more of the sporocyst. Capillitium sparse, purple-brown, curved, reticulate, with membranous enlargement. Spores dark in mass, violet-gray by transmitted light, (7–) 8–11 μm in diameter, large, with sparsely and irregularly warted.

**Habitat.** On the dead branches and fallen leaves.

**Distribution in China.** Jilin province, Liaoning province, Gansu province, Beijing City, Hebei province, Inner Mongolia Autonomous Region, Anhui province, Guangdong province, Heilongjiang province, Taiwan, Henan province, Jiangsu province.

**Distribution in the World.** Widely distributed.

**Specimens examined.** China, Jilin province, Jiaohe City, Lafashan National Forest Park, on the dead branches and fallen leaves, 6 September 2018, B. Zhang, HMJAU M10037. China, Liaoning province, Fuxin City, Haitangshan National Nature Reserve, on the branches and leaves, 1 September 2012, B. Zhang, HMJAU M10007. China, Gansu province, Tianshui City, Dangchuan Town forest farm, Maiji District, on the dead leaves and branches, 16 August 2010, B. Zhang, HMJAU M10002.

**Notes.** *Diachea bulbillosa* is morphologically similar to *D. splendens,* sharing features such as a subglobose sporocarp with a halo, long stalk, and conspicuous columella. However, *D. bulbillosa* differs from *D. splendens* by having larger, sparsely, and irregularly distributed warts, while *D. splendens* possesses thick tumorous processes, often connected into irregular lines or incomplete mesh.

***Diachea leucopodia*** (Bull.) Rostaf., *Sluzowce monogr*. 190 (1874).

Figure 6

**Figure 6:**
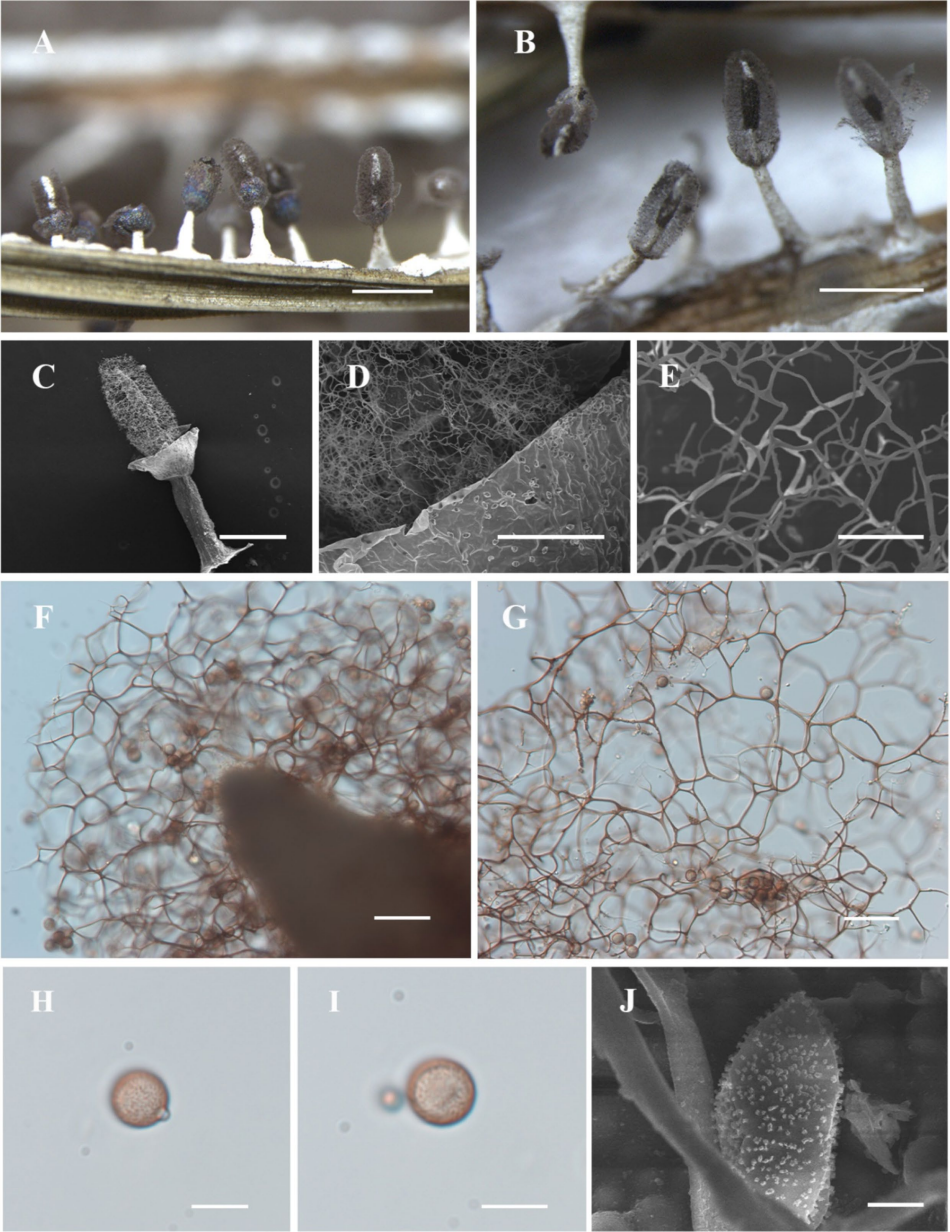
Habitat and microstructure of *Diachea leucopodia* (HMJAU M10005). (A,B) Sporocarps; (C) sporocarp by SEM; (D) capillitium and peridium by SEM; (E) capillitium by SEM; (F,G) columella and capillitium by TL; (H–J) spores with cluster warts by TL and SEM. Scale bars: (A), B = 1 mm, C = 400 μm, D = 100 μm, E = 20 μm, (F), G = 40 μm, (H), I = 10 μm, J = 2 μm.

**Description.** Sporocarp, gregarious or densely crowded, stipitate, metallic blue, or with a purple or bronze halo, cylindrical or elliptical, occasionally subspherical, with slightly concave navel below, (0.35–) 0.4–0.6 (−0.65) mm in diameter, (0.8–) 1–2 (−2.2) mm in tall. Stalk slender, calcareous, brittle, snow-white, approximately 1/2 the full height, tapering upward. Peridium often dehiscencing irregularly and falling off. Hypothallus white, calcareous, with veins and often forming a network. Columella thick, conical, gradually tapering upward, white, calcareous, extending more than half the height of the sporotheca, often reaching near the top of the sporotheca. Capillitium curved, branched, and interconnected, dark brown, colorless at the tips, radiating from various points of the columella. Spores dark in mass, light purple by transmitted light, rough, with minutely warted, 8–10 (−11) μm in diameter.

**Habitat.** On the dead branches and fallen leaves.

**Distribution in China.** Guangdong province, Guangxi Zhuang Autonomous Region, Yunnan province, Jilin province, Sichuan Province, Hebei province, Shanxi province, Inner Mongolia Autonomous Region, Jiangsu province, Fujian province, Shandong province, Hubei province, Shaanxi province, Gansu province, Henan province, Hunan province, Liaoning province, Taiwan, Hong Kong, Heilongjiang province, Beijing City, Ningxia Hui Autonomous Region, Anhui province.

**Distribution in the World.** Widely distributed.

**Specimens examined.** China, Guangdong province, Shaoguan City, Zhenjiang District, on the dead branches and fallen leaves, 13 May 2019, D. Dai, HMJAU M10086; China, Guangxi Zhuang Autonomous Region, Nanning City, Liang Feng Jiang National Forest Park, on the dead branches and fallen leaves, 15 August 2020, D. Dai, HMJAU M10087; China, Yunnan province, Lijiang City, on the dead branches and fallen leaves, 20 August 2012, B. Zhang, HMJAU M10005; China, Jilin province, Lushuihe Town, Changbai Mountain International Hunting Resort, on the dead branches and fallen leaves, 7 August 2014, B. Zhang, HMJAU M10015, HMJAU M10016; China, Jilin province, Jilin Agricultural University campus, on the dead branches and fallen leaves, 28 May 2013, B. Zhang, HMJAU M10018; China, Sichuan province, Ganzi Tibetan Autonomous Prefecture, Yajiang County, Gexigou Nature Reserve, on the dead leaves and branches, 5 August 2015, B. Zhang, HMJAU M10033.

**Notes.** *Diachea leucopodia* characterized by its white, calcareous hypothallus, stalk, and columella. The conical columella and cylindrical to oval sporocarps, along with light-color spores, and distinguish this species from others.

***Diachea macroverrucosa*** D. Dai & B. Zhang, *Mycology*: 51 (2024).

Figure 7

**Figure 7:**
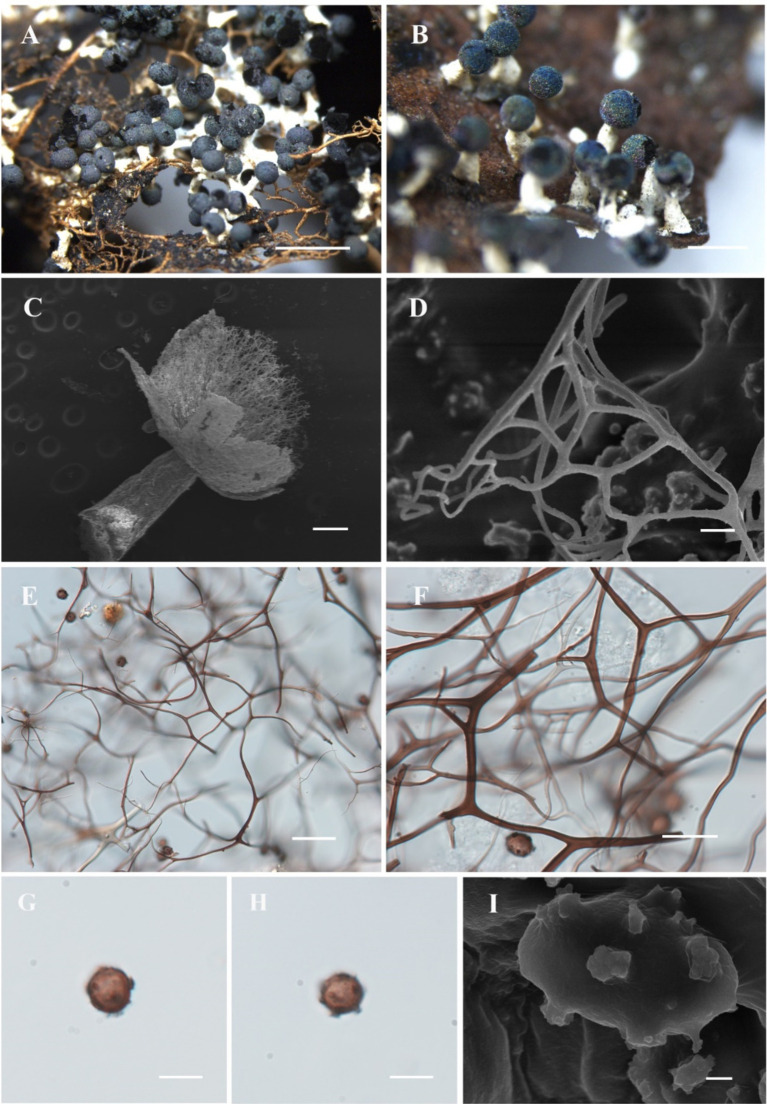
Habitat and microstructure of *Diachea macroverrucosa* (HMJAU M10064). (A,B) Sporocarps; (C) sporocarp by SEM; (D–F) capillitium by SEM and TL; (G–I) spores with sparse and large warts by TL and SEM. Scale bars: A = 2 mm, B = 1 mm, C = 100 μm, D = 5 μm, E = 40 μm, *F* = 20 μm, G, H = 10 μm, I = 1 μm.

**Description.** Sporocarp, gregarious to clusterous, stipitate, spherical, with haze and blue halo, (0.3–) 0.35–0.4 (−0.45) mm in diameter, (0.7–) 0.75–0.9 (−1) mm in tall. Stalk coarse and short, (0.35–) 0.4–0.5 (−0.55) mm in length, white, tapering upward, calcareous, containing numerous calcareous particles. Peridium single-layered, with a halo, dehiscencing irregularly. Hypothallus membranous, colorless, and transparent, sometimes forming sheets. Columella white, conical, blunt-headed, approximately 1/4 of the sporotheca. Capillitium dense, thin, hard, dark brown, branched, and interconnected, radiating from various points of the columella, lighter at the end, and not connected to the peridium. Spores black in mass, dark brown by transmitted light (TL), (8–) 9–13 μm in diameter, sparsely covered with large dark warts.

**Habitat**. On the branches and rotten leaves.

**Distribution in China**. Shaanxi province, Jilin Province, Liaoning Province.

**Distribution in the World**. China.

**Specimens examined, China**, Shaanxi province, Niubeiliang National Nature Reserve, on the rotten leaves, 21 July 2014, B. Zhang, HMJAU M10001; China, Jilin province, Yanbian Korean Autonomous Prefecture, Antu County, Changbai Mountain National Nature Reserve, on the rotten branches and leaves, 25 August 2013, B. Zhang, HMJAU M10027; China, Jilin province, Yanbian Korean Autonomous Prefecture, Longjing City, Tianfo Zhishan National Nature Reserve, on the rotten branches and leaves, 4 September 2011, B. Zhang, HMJAU M10009; China, Liaoning province, Fuxin City, Haitangshan National Nature Reserve, on the rotten branches and leaves, 1 September 2012, B. Zhang, HMJAU M10039, HMJAU M10057, HMJAU M10064, HMJAU M10067, HMJAU M10069, HMJAU M10073.

**Notes**. *Diachea macroverrucosa* is characterized by its large warts resembling an inflorescence on the spore surface. The columella is conical and does not exceed 1/2 the height of the sporocarp. Morphologically, *D. macroverrucosa* resembles *D. bulbillosa*, sharing characteristics such as sporangium shape, color, and spore diameter. Both species possess white columellae and stalks. However, *D. macroverrucosa* can be differentiated from *D. bulbillosa* by its shorter overall height and shorter columella, and sparse warts (Table 2).

***Diachea subsessilis*** Peck, Annual Rep. New York State Mus. 31:41 (1878).

Figure 8

**Figure 8:**
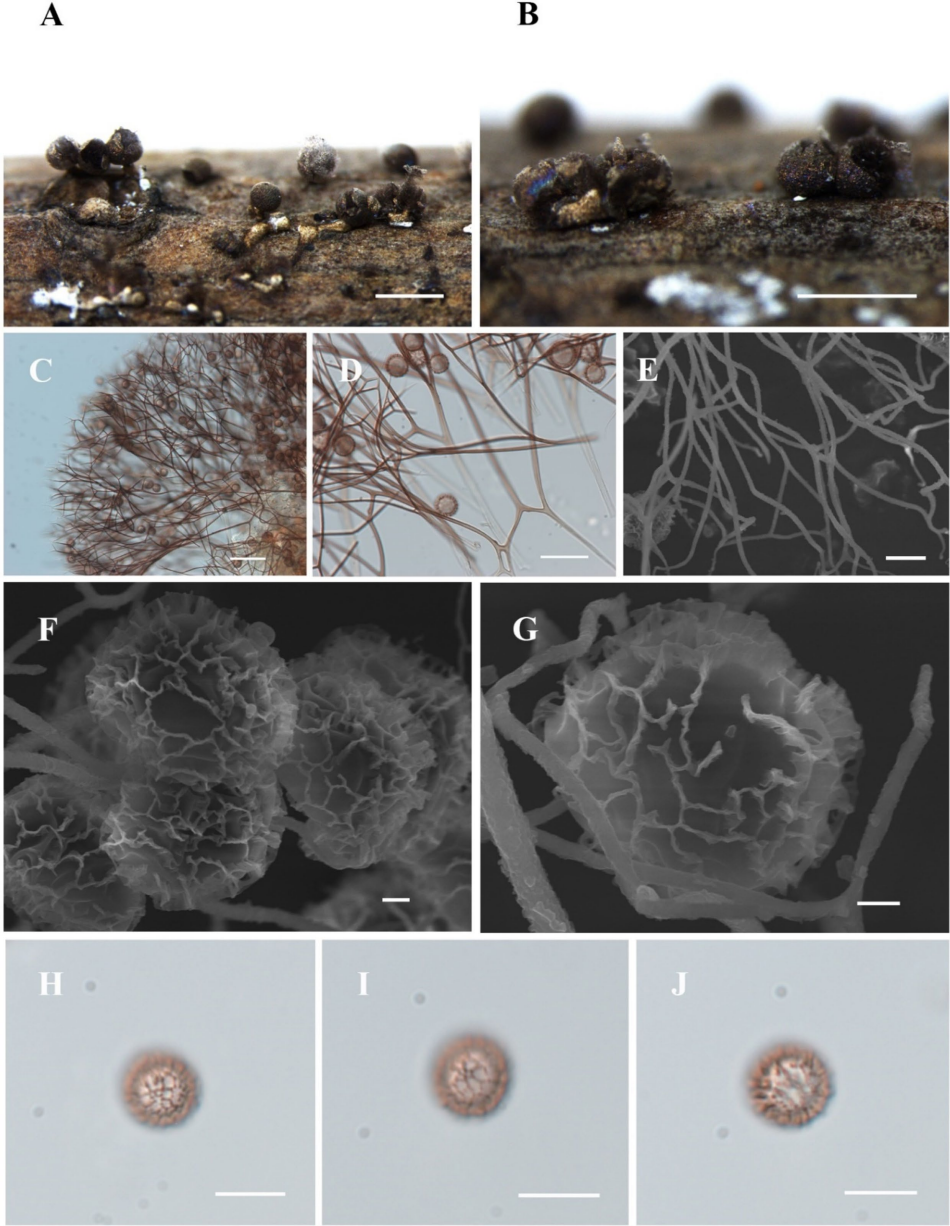
Habitat and microstructure of *Diachea subsessilis* (HMJAU M10075). (A,B) Sporocarps; (C–E) capillitium by TL and SEM; (F–J) Spores with clustered and connected into a network wart by SEM and TL. Scale bars: A, B = 1 mm, C = 40 μm, D = 20 μm, E = 5 μm, F, G = 1 μm, H-J = 10 μm.

**Description.** Sporocarp, gregarious or densely crowded, short-stalked or sessile, rarely plasmodiocarp, with gray-green or dark blue halo, (0.35–) 0.4–0.8 (−0.85) mm in diameter, (0.5–) 0.6–1 (−1.2) mm in tall. Stalk, when present, stout, cylindrical, white, calcareous, not exceeding the height of the sporocarp. Peridium single-layered, with halo. Hypothallus small, networked, slightly calcareous, or absent. Columella short, calcareous, conical, and sometimes absent. Capillitium thin, radiating from the columella, light brown, branched and connected, light-colored at the ends. Spores dark in mass, light brown by transmitted light, with clustered warts and lines connected into a network, (7–) 8–12 μm in diameter.

**Habitat.** On the branches and rotten leaves.

**Distribution in China.** Sichuan province, Guangxi Zhuang Autonomous Region, Jiangsu province.

**Distribution in the World.** China, North America, Europe, and Southeast Asia.

**Specimens examined.** China, Sichuan province, Chengdu city, Tazishan Park, on the branches and rotten leaves, 15 July 2013, B. Zhang, HMJAU M10031, HMJAU M10075.

**Notes.** *Diachea subsessilis* is characterized by its short-stalked or sessile sporocarp with a gray-green or dark blue halo. It shares morphological similarities with *D. radiata* including a subglobose sporocarp, similar spore size, white columella, and stalk. However, *D. subsessilis* can be differentiated by its short columella. The spores have a spinulose-reticulate surface, often forming a complete net on one side, whereas *D. radiata* has small spinulose warts.

**Taxonomic key to the species of *Diachea.***

1. Capillitium with limestone………………………………………………………*Diachea dictyospora.*

1. Capillitium without limestone…………………………………………….…………………………2.

2. Hypothallus, stalk, and columella are orange……………………………..… *Diachea silvaepluvialis.*

2. Hypothallus, stalk, and columella are white………………………………………………..….……3.

3. Sporocarp generally cylindrical or oval, rarely subglobose…………………………..…..….…….4.

3. Sporocarp spherical…………………………………………………………………………….…….5.

4. Spores are light in color and nearly smooth………………..………………..….*Diachea leucopodia.*

4. Spores are light in color, with warts and dark clusters…….……………….…*Diachea sichuanensis.*

5. Spores with reticular patterns and capillitium are soft and slender………………*Diachea subsessilis.*

5. Spores with warts or nodular protrusion…………………….………………………………………6.

6. Spores with less nodular protrusion, large…………………………………*Diachea macroverrucosa.*

6. Spores with more nodular protrusion, small…………………………………………………………7.

7. Capillitium are sparse and curly………………………………………………..…*Diachea bulbillosa.*

7. Capillitium dense, hard, and straight………………….……………………..….*Diachea plectophylla.*

## 4 Discussion

This study significantly advances our understanding of *Diachea* by providing detailed descriptions of two newly identified species, namely, *D. plectophylla* and *D. sichuanensis*, supported by robust phylogenetic analysis. In addition, we report new distribution records for five previously known species: *D. bulbillosa* in Gansu province, *D. leucopodia* in Yunnan and Sichuan provinces, and *D. subsessilis* in Sichuan province, China. Morphologically, *D. plectophylla* resembles *D. bulbillosa* with its sparse warts, iridescent sporocarps, white stalk, and prominent columella. However, it differs in having smaller spores and occasionally interconnected warts forming ridges. *D. sichuanensis* can be distinguished from *D. leucopodia* by its blunt-headed columella, spores with clustered warts, and thicker stalks. A detailed comparison of species characteristics is provided in Table 2.

Traditionally, species identification in myxomycete has relied on morphological traits. However, advances in molecular biology have greatly improved the accuracy of species identification, helping resolve discrepancies caused by morphology alone. Our morphological distinctions are further validated by the unique placements of these species, which confirms their taxonomic status. In our phylogenetic analysis, *Diachea* species formed a distinct branch within the family *Physaraceae*, contrasting with some recent research (Prikhodko et al., 2023). Studies by Fiore-Donno et al. (2012) and Cainelli et al. (2020) suggested that *Diachea* and *Didymium* are sister branches, albeit with weak support. Our results, however, suggest that *Diachea*, *Nannengaella*, and *Physarum* are more closely related sister branches.

Lado et al. (2022), who used the 18S rRNA, 17S rRNA, and *EF-1α* genes, faced challenges with incomplete data, leading to weak support in the ML analysis for six species. Discrepancies between our study and previous research may arise from the use of different gene markers. For instance, García-Martín et al. (2023) redefined *Diachea* to include species previously classified in *Physaraceae*, such as *Craterium* spp., but placed *Diachea* within *Didymiaceae*, divided into two groups, which diverges from our findings. Similarly, Prikhodko et al. (2023) used three gene markers to analyze *Didymiaceae*, resulting in some reclassifications. We believe that these differences stem from the varying gene markers, sample size, and available sequences used in each study. In addition, factors such as collection area, climate, and vegetation type may have contributed to the variations observed.

The taxonomic classification of the genus *Diachea* has historically been contentious. Despite its wide geographical distribution and the growing number of reported species, the limited availability of specimens and genetic sequences has hindered accurate classification. The scarcity of known *Diachea* sequences, along with unidentified sequences uploaded to NCBI, may lead to misinterpretations when using BLAST search. In recent studies, *Diachea* has been classified within the order Physarales; but its placement has oscillated between the families *Physaraceae* (Lister, 1925) and *Didymiaceae* (Keller and Braun, 1999; García-Martín et al., 2023; Prikhodko et al., 2023).

Our research identified five species forming a distinct evolutionary branch, which we tentatively assigned to the family *Didymiaceae* based on recent systematic research on Physarales (García-Martín et al., 2023). However, due to the limited number of specimens, we did not conduct a comprehensive analysis of the entire genus, focusing instead on the discovery of two new species. The limited data leave their taxonomic status somewhat uncertain. Nonetheless, our findings provide valuable sequences and preliminary insights for future systematic studies of this genus.

The recent discovery of new *Diachea* species, such as *D. mitchellii* (Lado et al., 2022), *D. racemosa* (Novozhilov et al., 2023), and *D. macroverrucosa* (Liu et al., 2024), underscores the greater species richness within the genus than previously documented. Looking ahead, we plan to expand our sampling range, enhance morphological observations, increase the number of sequences, and employ additional gene markers to deepen our understanding of the relationships among *Diachea*, *Didymiaceae*, and *Physaraceae*. Future research should incorporate more gene markers (nSSU, *EF-1α*, mtSSU, *COI*, and *α-Tub*) and aim to obtain complete sequences of these genes for a more comprehensive study. To minimize variations caused by regional and environmental factors, collaboration with international researchers to broaden the sampling area is recommended. In addition, increasing specimen collection from extreme environments will facilitate a more thorough and systematic study of this genus and its relatives, ultimately clarifying its taxonomic status.

## 5 Conclusion

This study identifies two newly discovered species of *Diachea* and provides a detailed morphological description of these species, along with five previously known ones. An identification key for these species is also included. Given the limited research and sequence data available for this genus, our findings offer valuable foundational information to support future systematic research. Furthermore, the discovery of these new and newly recorded species enhances our understanding of *Diachea* diversity, contributing to broader research on the community composition ecological roles, and spatiotemporal distribution of myxomycetes and their substrates.

## Data Availability

The datasets presented in this study can be found in https://www.ncbi.nlm.nih.gov/nuccore. The accession number(s) can be found in the article/supplementary material.

## Footnotes

^1^http://www.ncbi.nlm.nih.gov/genbank
